# Diagnostic and Predictive Contribution of Autoantibodies Screening in a Large Series of Patients With Primary Immunodeficiencies 

**DOI:** 10.3389/fimmu.2021.665322

**Published:** 2021-04-01

**Authors:** Azzeddine Tahiat, Abdelghani Yagoubi, Mohamed Samir Ladj, Reda Belbouab, Samira Aggoune, Laziz Atek, Djamila Bouziane, Souhila Melzi, Chahinez Boubidi, Warda Drali, Chafa Bendahmane, Hamza Iguerguesdaoune, Sihem Taguemount, Asma Soufane, Asma Oukil, Abdalbasset Ketfi, Hassen Messaoudi, Nadia Boukhenfouf, Mohamed Amine Ifri, Tahar Bencharif Madani, Hayet Belhadj, Keltoum Nafissa Benhala, Mokhtar Khiari, Nacera Cherif, Leila Smati, Zakia Arada, Zoulikha Zeroual, Zair Bouzerar, Ouardia Ibsaine, Hachemi Maouche, Rachida Boukari, Kamel Djenouhat

**Affiliations:** ^1^ Department of Medical Biology, Rouiba Hospital, Algiers Faculty of Medicine, University of Algiers 1, Algiers, Algeria; ^2^ Pediatric Gastroenterology, Centre Algérois de Pédiatrie, Algiers, Algeria; ^3^ Department of Pediatrics, Mustapha University Hospital, Algiers Faculty of Medicine, University of Algiers 1, Algiers, Algeria; ^4^ Department of Pediatrics, El-Harrach Hospital, Algiers Faculty of Medicine, University of Algiers 1, Algiers, Algeria; ^5^ Department of Pediatrics, Ain Taya Hospital, Algiers Faculty of Medicine, University of Algiers 1, Algiers, Algeria; ^6^ Department of Pediatrics, Bab El-Oued University Hospital, Algiers Faculty of Medicine, University of Algiers 1, Algiers, Algeria; ^7^ Department of Pediatrics A, Hussein Dey University Hospital, Algiers Faculty of Medicine, University of Algiers 1, Algiers, Algeria; ^8^ Department of Pediatrics B, Hussein Dey University Hospital, Algiers Faculty of Medicine, University of Algiers 1, Algiers, Algeria; ^9^ Department of Pediatrics, Meftah Hospital, Blida, Algeria; ^10^ Department of Pneumology, Rouiba Hospital, Algiers Faculty of Medicine, University of Algiers 1, Algiers, Algeria; ^11^ Department of Internal Medicine, Mustapha University Hospital, Algiers Faculty of Medicine, University of Algiers 1, Algiers, Algeria; ^12^ Department of Pediatrics, Rouiba Hospital, Algiers, Algeria; ^13^ Department of Pediatrics, Thenia Hospital, Boumerdes, Algeria; ^14^ Department of Pediatrics, Mansourah Hospital, Constantine, Algeria; ^15^ Department of Pediatrics, Central Hospital of the Army, Algiers, Algeria; ^16^ Department of Pediatrics A, Beni Messous University Hospital, Algiers Faculty of Medicine, University of Algiers 1, Algiers, Algeria; ^17^ Department of Pediatrics B, Beni Messous University Hospital, Algiers Faculty of Medicine, University of Algiers 1, Algiers, Algeria; ^18^ Department of Pediatrics, Bologhine Hospital, Algiers Faculty of Medicine, University of Algiers 1, Algiers, Algeria

**Keywords:** primary immunodeficiencies, autoantibody, screening, autoimmune cytopenia, celiac disease, platelet-bound IgM, transglutaminase antibody

## Abstract

**Objectives:**

To evaluate the diagnostic and predictive contribution of autoantibodies screening in patients with primary immunodeficiencies (PIDs).

**Methods:**

In the present study, PID patients and healthy controls have been screened for 54 different autoantibodies. The results of autoantibodies screening in PID patients were correlated to the presence of autoimmune diseases.

**Results:**

A total of 299 PID patients were included in this study with a predominance of antibody deficiencies (27.8%) followed by immunodeficiencies affecting cellular and humoral immunity (26.1%) and complement deficiencies (22.7%). Autoimmune manifestations were present in 82 (27.4%) patients. Autoimmune cytopenia (10.4%) was the most common autoimmune disease followed by gastrointestinal disorders (10.0%), rheumatologic diseases (3.7%), and endocrine disorders (3.3%). Autoantibodies were found in 32.4% of PID patients and 15.8% of healthy controls (*P* < 0.0005). Anti-nuclear antibodies (ANA) (10.0%), transglutaminase antibody (TGA) (8.4%), RBC antibodies (6.7%), anti-smooth muscle antibody (ASMA) (5.4%), and ASCA (5.0%) were the most common autoantibodies in our series. Sixty-seven out of the 82 patients with autoimmune manifestations (81.7%) were positive for one or more autoantibodies. Eleven out of the 14 patients (78.6%) with immune thrombocytopenia had positive platelet-bound IgM. The frequencies of ASCA and ANCA among patients with IBD were 47.4% and 21.0% respectively. All patients with celiac disease had TGA-IgA, while six out of the 11 patients with rheumatologic diseases had ANA (54.5%). Almost one third of patients (30/97) with positive autoantibodies had no autoimmune manifestations. ANA, rheumatoid factor, ASMA, anti-phospholipid antibodies and ANCA were often detected while specific AID was absent. Despite the low positive predictive value of TGA-IgA and ASCA for celiac disease and inflammatory bowel disease respectively, screening for these antibodies identified undiagnosed disease in four patients with positive TGA-IgA and two others with positive ASCA.

**Conclusion:**

The present study provides valuable information about the frequency and the diagnostic/predictive value of a large panel of autoantibodies in PIDs. Given the frequent association of some AIDs with certain PIDs, screening for corresponding autoantibodies would be recommended. However, positivity for autoantibodies should be interpreted with caution in patients with PIDs due to their low positive predictive value.

## Introduction

Primary immunodeficiencies (PIDs) are a heterogeneous group of genetic disorders that affect distinct components of both humoral and cellular arms of the immune system (1). PIDs are no longer defined by recurrent infections alone. Patients with such disorders are increasingly recognized with features of immune dysregulation, including autoimmunity and inflammation (2–5). In a retrospective study of the French Registry, authors have reported that more than 26% of patients with PIDs developed one or more autoimmune or inflammatory manifestations throughout their lifetime (6). The risk for autoimmune diseases (AID) was at least 10 times higher than in the general population (6). Mechanisms underlying the development of autoimmunity in PIDs include defective T and B cell development and tolerance, defective regulatory T cell (Treg) development or function, increased type I interferon signature and lack of clearance of immune complexes and apoptotic debris (7).

The presence of serum autoantibodies directed to multiple cell surface and intracellular antigens is a serological hallmark of autoimmune diseases and a helpful biomarker for establishing an early and accurate diagnosis. Autoimmunity has been widely studied in PIDs. However, most of published studies were focused on clinical manifestations and only few is known about the clinical relevance of autoantibody testing in such monogenic defects (6, 8). In the present study, a large series of patients with PIDs have been screened for a broad panel of autoantibodies. The study’s main objective was to evaluate the diagnostic and predictive contribution of autoantibodies screening in patients with PIDs. A secondary goal was to report the frequency of autoantibodies in different PID categories.

## Material and Methods

### Patients and Healthy Controls

In the present study, 299 Algerian patients with PIDs have been enrolled in two-year period (January 2018 to January 2020). All patients met the updated criteria of the European Society for Immunodeficiency (ESID) (www.esid.org). Secondary immunodeficiencies were ruled out for each patient. Patients were categorized according to the International Union of Immunological Societies (IUIS), Primary Immunodeficiency Diseases Committee Report on Inborn Errors of Immunity (2019) (1). In addition, the study included 120 healthy subjects (70 children and 50 adults) as a control group. The study was approved by the local ethics Committee and it conforms to the provisions of the World Medical Association’s Declaration of Helsinki.

### Diagnosis of Autoimmune Diseases

Data on autoimmune manifestations were collected and analyzed for each patient. The diagnosis of AID was based on clinical and complementary paraclinical findings (ex., radiology, endoscopy, colonoscopy and biopsy results) and laboratory tests (ex., Coombs test, antinuclear antibodies, and transglutaminase antibody), according to international criteria for a specific disease. For instance, patients who were diagnosed with autoimmune hemolytic anemia (AIHA) presented with anemia that was associated with a positive Coombs test.

### Autoantibody Testing

Patients and healthy controls were systematically screened for a broad panel of autoantibodies (54 distinct autoantibodies) that included : anti-nuclear antibodies (ANA), rheumatoid factor (RF), anti-citrullinated protein antibody (ACPA), anti-neutrophil cytoplasmic antibodies (ANCA), anti-phospholipid antibodies (aPL), anti-tissue antibodies (such as anti-smooth muscle antibody (ASMA)), anti-saccharomyces cerevisiae antibody (ASCA), transglutaminase antibody (TGA), red blood cell (RBC) antibodies, platelet-bound antibodies (PA) and anti-neutrophil antibodies (**Table 1**).

**Table 1:** Immunoassays used for antibody testing.

AID	Autoantibody	Immunoassay (manufacturer)
**Rheumatologic diseases**	ANA (screening)	IIF/HEp-2 (EUROIMMUN, Germany)
	ANA (identification) dsDNA ENA : Sm, RNP, Sm/RNP, SSA, SSB, Scl-70, PM/Scl, Ku, CENP-A/B, PCNA, Mi-2 & DFS-70 Nucleolar antigens : PM/Scl, Th/To, Fibrillarin, NOR-90 Cytoplasmic antigens : M2/nPDC, Jo-1, PL-7, PL-12, SRP and Ribosome P0	ELISA (Bio-Rad, CA, USA) Immunodot (D-tek, Belgium) Immunodot (D-tek, Belgium) Immunodot (D-tek, Belgium)
	RF	Laser Nephelometry (BN ProSpec, Siemens, Germany)
	ACPA	ELISA (Bio-Rad, CA, USA)
**APS**	aCL-IgG & aCL-IgM	ELISA (Bio-Rad, CA, USA)
	aβ2GPI-IgG & β2GPI-IgM	ELISA (Bio-Rad, CA, USA)
**AAV**	ANCA (screening)	IIF/human neutrophils (EUROIMMUN, Germany)
	ANCA (identification)	Immunodot (D-tek, Belgium)
**IBD**	ASCA-IgA & ASCA-IgG	ELISA (Bio-Rad, CA, USA)
**CD**	TGA-IgA & TGA-IgG	ELISA (Bio-Rad, CA, USA)
**Pernicious anemia**	APCA	IIF (EUROIMMUN, Germany)
**AIH & PBC**	ASMA	IIF (EUROIMMUN, Germany) and Immunodot (D-tek, Belgium)
	anti-LKM 1	IIF (EUROIMMUN, Germany) and Immunodot (D-tek, Belgium)
	anti-LC1	IIF (EUROIMMUN, Germany) and Immunodot (D-tek, Belgium)
	anti-SLA	Immunodot (D-tek, Belgium)
	AMA	IIF (EUROIMMUN, Germany) and Immunodot (D-tek, Belgium)
	anti-gp210	Immunodot (D-tek, Belgium)
	anti-SP100	Immunodot (D-tek, Belgium)
**Autoimmune thyroiditis**	anti-TPO	ELISA (EUROIMMUN, Germany)
	anti-TG	ELISA (EUROIMMUN, Germany)
**Insulin-dependent diabetes mellitus**	anti-GAD	ELISA (EUROIMMUN, Germany)
**AIHA**	RBC antibodies	Coombs test
**ITP**	PA (IgG, IgM et IgA)	Flow cytometry
**Autoimmune neutropenia**	anti-neutrophil antibodies (IgG et IgM)	Indirect GIFT- Flow cytometry

AAV, ANCA associated vasculitis; ACPA, anti-citrullinated protein antibody; aCL, anti-cardiolipin antibodies; aβ2GPI, anti-β2 glycoprotein I antibodies; AID, autoimmune disease; AIH, autoimmune hepatitis; AIHA, autoimmune hemolytic anemia; AMA, anti-mitochondrial antibody; ANA, anti-nuclear antibodies; ANCA, anti-neutrophil cytoplasmic antibodies; APCA, anti-parietal cell antibody; APS, anti-phospholipid syndrome; ASCA, anti-saccharomyces cerevisiae antibody; ASMA, anti-smooth muscle antibody; CD, celiac disease; ELISA, enzyme-like immunosorbent assay, GIFT, granulocyte immunofluorescence test; IBD: inflammatory bowel disease; IIF, indirect immunofluorescence, ITP, immune thrombocytopenia; LC, liver cytosol; LKM, liver-kidney microsome; PA, platelet-bound antibodies; PBC, primary biliary cirrhosis; RBC, red blood cell; RF, rheumatoid factor; SLA, soluble liver antigen; TG, thyroglobulin; TGA, anti-transglutaminase antibody; TPO, thyroid peroxidase.

ANA screening was performed at a dilution of 1:80 by indirect immunofluorescence (IIF) using HEp-2 cell line as substrate. For positive sera, nuclear and cytoplasmic staining patterns were read by two experts and ANA specificities, including dsDNA, extractable nuclear antigens (ENA), nucleolar antigens and cytoplasmic antigens were identified by enzyme-like immunosorbent assay (ELISA) and/or Immunodot. IIF and ELISA have been used for ANCA screening and identification, respectively. Detection of RBC antibodies was performed by Coombs test. PA-IgG, PA-IgM and PA-IgA were detected by flow cytometry, using a commercial kit (THROMBOCYTEST, BD Biosciences, CA, USA), according to the manufacturer’s instructions. Briefly, isolated platelets from EDTA whole blood were incubated with PE-labeled goat Ig anti-human IgG, IgM and IgA to detect platelet-bound antibodies. Binding of the antibodies was measured with a FACSCanto (BD Biosciences, CA, USA). Anti-neutrophil antibodies (IgG and IgM) were detected by indirect granulocyte immunofluorescence test (GIFT) as described previously (9). Briefly, a mixture of fresh anticoagulated blood samples obtained from 10 healthy subjects was used as a source of neutrophils. The rationale for using multiple donors was to obtain a good representation of two common human neutrophil antigens (HNA) alloforms 1 and 2. The mixed blood sample was treated with ammonium chloride-based RBC-lysing reagent. The cells were thoroughly washed, resuspended and incubated at room temperature with the patients’ sera for 30 minutes, washed twice and incubated for 15 minutes with FICT-conjugated anti-human IgG and IgM rabbit F(ab’)_2_ antibody fragment.

### Statistical Analysis

The comparison of frequencies was performed by chi-square test or Fisher’s exact test. The threshold for statistical significance was set to a *P* value of less than 0.05 in all analyses. The statistical analyses were performed using SPSS version 23.0 (software package (IBM), Chicago, IL, USA).

## Results

### Demographic Data

The demographic data of PID patients and healthy controls are shown in **Table 2**. Of the 299 patients included in this study, 169 (56.5%) were males and 130 (43.5%) were females. The mean age of patients was 12.8 years (0.1 – 80 years), the mean age at onset of PID symptoms was 4,2 years (0 – 60 years) and the mean disease duration was 5.7 years (0.1 – 53 years). Two hundred three (68%) patients were children and 96 (32%) were adults. Ninety patients (30.1%) were born from consanguineous parents. Diagnosis of PID was confirmed by immunological testing in all patients and genetically in 60 (20%) patients. The distribution of patients according to the classification from the IUIS Expert Committee is shown in **Table 2**. Predominantly antibody deficiencies (27.8%) were the most common, followed by immunodeficiencies affecting cellular and humoral immunity (26.1%) and complement deficiencies (22.7%).

**Table 2:** Demographic data of PID patients and healthy controls.

Category	Number (%)	Gender M/F	Age Mean/years (range)	Disease duration Mean/years (range)
Immunodeficiencies affecting cellular and humoral immunity	78 (26.1)	48/30	3.9 (0.1 – 76)	2.8 (0 – 21)
Combined immunodeficiencies with associated or syndromic features	28 (9.4)	20/8	3.3 (0.2 – 14)	3.0 (0 – 13)
Predominantly antibody deficiencies	83 (27.8)	46/37	19.2 (0.3 – 80)	6.6 (0 – 30)
Diseases of immune dysregulation	20 (6.7)	13/7	4.4 (0.3 – 17)	3.4 (0 – 13)
Congenital defects of phagocyte number or function	9 (3.0)	7/2	4.8 0.1 – 31	0.7 (0 – 2)
Defects in intrinsic and innate immunity	6 (2.0)	2/4	6.7 (2 – 21)	5.7 (1.5 – 17)
Complement deficiencies	68 (22.7)	28/40	26.6 (0.5 – 67)	18.7 (2 – 53)
Other immunodeficiencies	7 (2.3)	5/2	20.1 2 – 65	1.1 (1 – 3)
Healthy controls	120	69/51	17.9 (1 – 58)	/

### Autoimmune Diseases

Autoimmune manifestations were present in 82 (27.4%) patients, including 45 (55%) males and 37 (45%) females. There was no statistically significant association between having AID and gender (*P* = 0.72). AIDs was more common in patients with immune dysregulation (70.0%), followed by immunodeficiencies affecting cellular and humoral immunity (33.3%) (*P* < 0.0005). The lowest frequency of AID was seen in patients with complement deficiencies (10.3%) (**Table 3**). Unlike patients with “classical” severe combined immunodeficiencies (SCIDs), patients with atypical forms of SCID, including Omenn syndrome (OS) (*n* = 6) and leaky SCID (*n* = 9) presented a high frequency of AID (14.3% *vs.* 66.7%; *P =* 0,006). The frequency of autoimmune manifestations in patients with predominantly antibody deficiencies was 24.1%. Such manifestations were more frequent in patients with common variable immunodeficiencies (CVID) compared to patients with other antibody deficiencies (30.9% *vs.* 17.1%), but the difference did not reach level of significance (*P* = 0.14) (**Tables 3** and **5**).

**Table 3:** AID and autoantibodies in different PID categories and healthy controls.

Category	AIDs*n(%)*	Autoantibodies*n(%)*
Immunodeficiencies affecting cellular and humoral immunity	26 (33.3)	30 (38.5)
Combined immunodeficiencies with associated or syndromic features	8 (28.6)	14 (50.0)
Predominantly antibody deficiencies	20 (24.1)	17 (20.5)
Diseases of immune dysregulation	14 (70.0)	13 (65.0)
Congenital defects of phagocyte number or function	3 (33.3)	3 (33.3)
Defects in intrinsic and innate immunity	2 (33.3)	2 (33.3)
Complement deficiencies	7 (10.3)	15 (22.1)
Healthy controls	0 (0)	19 (15.8)

AID, autoimmune disease.

A wide range of autoimmune manifestations was observed in our series; autoimmune cytopenia (10.4%) was the most common AID followed by gastrointestinal disorders (10.0%), rheumatologic diseases (3.7%), skin manifestations (3.3%) and endocrine disorders (3.3%) (**Table 4**). The distribution of different AIDs with respect to PID categories is shown in **Table 5**. The most striking findings are that autoimmune cytopenia and skin diseases were common in patients with combined immunodeficiencies (CID), especially atypical SCID, while IBD and endocrine disorders predominated in patients with diseases of immune dysregulation. IBD was also common in patients with chronic granulomatous disease (CGD).

**Table 4:** Main autoimmune manifestations in our series.

AID	Number	Percentage (*n* = 299)
Autoimmune cytopenia	31	10,4
AIHA	17	5,7
ITP	11	3,7
Evans syndrome	3	1,0
Autoimmune neutropenia	3	1,0
Gastrointestinal disorders	30	10.0
IBD	19	6,4
Celiac disease	8	2,7
Pernicious anemia	4	1.3
Rheumatologic disorders	11	3,7
SLE	4	1.3
Endocrine disorders	10	3,3
Insulin-dependent diabetes mellitus	6	2,0
Hashimoto’s disease	4	1,3
Skin diseases	10	3,3

AID, autoimmune disease; AIHA, autoimmune hemolytic anemia; IBD: inflammatory bowel disease; ITP, immune thrombocytopenia; SLE, systemic lupus erythematosus.

**Table 5:** Distribution of major autoimmune diseases among different categories/types of PIDs.

Category/type of PIDs	Number of patients	Autoimmune Cytopenia(%)	IBD(%)	CD(%)	Endocrine disease(%)	Skin(%)
Immunodeficiencies affecting cellular and humoral immunity	78	17.9	2.6	2.6	1.3	10.3
SCID	14	14.3	0	0	0	0
OS	6	50.0	0	0	0	100
Leaky SCID	9	33.3	0	0	0	11.1
*bona fide* CID	49	12.2	4.1	4.1	2.0	2.0
Combined immunodeficiencies with associated or syndromic features	28	17.9	0	3.6	3.6	0
Wiskott–Aldrich syndrome	6	33.3	0	16.7	0	0
DiGeorge syndrome	4	25.0	0	0	0	0
Ataxia telangiectasia	6	0	0	0	0	0
Hyper-IgE syndrome	7	0	0	0	0	0
Other CID with syndromic features	5	40	0	0	20	0
Predominantly antibody deficiencies	83	8.4	7.2	2.4	1.2	1.2
Agammaglobulinemia	14	0	0	0	0	0
CVID	42	11.9	9.5	4.8	0	2.4
Other antibody deficiencies	27	7.4	7.4	0	3.7	0
Diseases of immune dysregulation	20	20.0	40.0	5.0	20.0	5.0
ALPS (Fas deficiency)	3	100	0	0	0	0
IPEX syndrome	1	100	0	0	100	0
LRBA deficiency	1	0	0	0	0	0
Immune dysregulation with colis	6	0	100	0	0	16.7
Familial hemophagocytic lymphohistiocytosis	3	0	0	0	0	0
Chediak Higashi	2	0	0	0	0	0
Other diseases of immune dysregulation	4	0	50	25	75	0
Congenital defects of phagocyte number or function	9	11.1	22.2	0	11.1	0
CGD	4	0	50.0	0	25.0	0
Congenital neutropenia	3	33.3	0	0	0	0
Other phagocytic defects	2	0	0	0	0	0
Defects of intrinsic and innate immunity	6	0	0	0	16.7	0
STAT1 GOF	2	0	0	0	50	0
MSMD	4	0	0	0	0	0
Complement deficiencies	68	0	0	2.9	0	0
HAE/C1-inhibitor deficiency	55	0	0	3.6	0	0
Other complement deficiencies	13	0	0	0	0	0

ALPS, autoimmune lymphoproliferative syndrome; CD, celiac disease; CGD, chronic granulomatous disease; CID, combined immunodeficiency; CVID, common variable immunodeficiency; GOF, gain-of-function; HAE, hereditary angioedema; IBD, inflammatory bowel disease; IPEX, immune dysfunction, polyendocrinopathy, enteropathy, LRBA, lipopolysaccharide-responsive and beige-like anchor protein; MSMD, mendelian susceptibility to mycobacterial disease; OS, Omenn Syndrome; PID, primary immunodeficiency disease; SCID, severe combined immunodeficiency; STAT, signal transducer and activator of transcription.

### Autoantibodies

The results of autoantibody testing in PID patients and healthy controls are shown in **Table 3** and **6**. One or more autoantibodies were found in 32.4% of PID patients and 15.8% of healthy individuals (*P* < 0.0005). Among the 97 patients with positive autoantibodies, 58 (60%) were males and 39 (40%) were females. There was no statistically significant association between having autoantibodies and gender (*P* = 0.54). The distribution of patients according to the number of positive autoantibodies showed that 46 patients (47.4%) were positive for 1 autoantibody, 22 patients (22.7%) were positive for 2 autoantibodies, 15 patients (15.5%) had 3 autoantibodies, while 14 patients (14.4%) developed 4 or more autoantibodies. There was a statistically significant association between having autoantibodies and PID category (*P* = 0.001). Serum autoantibodies were more frequent in patients with diseases of immune dysregulation followed by T cell immunodeficiencies, including immunodeficiencies affecting cellular and humoral immunity (38.5%) and CID with associated or syndromic features (50%) (**Table 3**).

**Table 6:** Frequencies of different autoantibodies in PID patients and healthy controls.

Autoantibody	PID patients *%*	Healthy controls *%*	*P*
Autoantibodies	32.4	15.8	<0.0005
RBC antibodies	6.7	0	0.002
PA	4.3	0	0.024
PA-IgM	4.0	0	0.023
PA-IgG	1.7	0	NS
PA-IgA	0.7	0	NS
aNL	1.0	ND	ND
aNL-IgM	0.7	ND	ND
aNL-IgG	0.3	ND	ND
ASCA	5.0	4.2	NS
ASCA-IgA	4.3	4.2	NS
ASCA-IgG	1.3	1.7	NS
ANCA	4.3	0.8	NS
TGA	8.4	1.7	0.014
TGA-IgA	7.4	1.7	0.021
TGA-IgG	2.3	0.8	NS
APCA	2.0	2.5	NS
ANA	10.0	6.7	NS
anti-dsDNA	1.7	0	NS
anti-ENA	3.7	0	0.039
anti-SSA/Ro	1.3	0	NS
anti-SSB/La	0.7	0	NS
anti-RNP	1.3	0	NS
anti-Sm	0.7	0	NS
anti-Scl70	0.3	0	NS
anti-Ku	0.3	0	NS
anti-DFS70	0.3	0	NS
RF	5.0	0.8	0.048
ACPA	1.0	0.0	NS
ASMA	5.4	0.8	0.051
anti-LKM1	0.3	0.0	NS
anti-GAD	1.7	ND	ND
anti-TPO	2.0	ND	ND
anti-TG	1.3	ND	ND
aPL	2.7	0.8	NS
aCL-IgG	1.0	0	NS
aCL-IgM	1.0	0.8	NS
aβ2GPI-IgG	0	0	NS
aβ2GPI-IgM	1.0	0	NS

ACPA, anti-citrullinated protein antibody; aCL, anti-cardiolipin antibodies; aβ2GPI, anti-β2 glycoprotein I antibodies; ANA, anti-nuclear antibodies; ANCA, anti-neutrophil cytoplasmic antibodies; aNL, anti-neutrophil antibody; APCA, anti-parietal cell antibody; aPL, antiphospholipid antibodies; ASCA, anti-saccharomyces cerevisiae antibody; ASMA, anti-smooth muscle antibody; GAD, anti-glutamic acid decarboxylase, LKM, liver-kidney microsome; ND, not determined; NS: not significant; PA, platelet-bound antibodies; RBC, red blood cell; RF, rheumatoid factor; TG, thyroglobulin; TGA, anti-transglutaminase antibody; TPO, thyroid peroxidase.

The most frequent autoantibodies in our series were: ANA (10.0%), TGA (8.4%), RBC antibodies (6.7%), ASMA (5.4%), RF (5.0%), ASCA (5.0%), ANCA (4.3%), and PA (4.3%). RBC antibodies (*P* = 0.002), PA-IgM (*P* = 0.023), TGA-IgA (*P* = 0.021), anti-ENA antibodies (*P* = 0.039), and RF (*P* = 0.048) were significantly more frequent in PID patients when compared to healthy controls (**Table 6**). The distribution of different autoantibodies with respect to PID categories is shown in **Table 7**. The frequencies of most tested autoantibodies were higher in patients with diseases of immune dysregulation (**Table 7**). However, when considering PID patients with increased susceptibility to infections, and after excluding patients who have defects of phagocyte number or function, and defects in intrinsic and innate immunity due to low numbers, the frequency of RBC antibodies was significantly higher in patients with T cell immunodeficiency (*P* = 0.003). The frequency of PA was also higher in patients with T cell immunodeficiency but the difference did not reach level of significance (*P* = 0.26). There was no statistically significant association between having ANA, ASCA, ANCA and TGA-IgA and PID category. However, it is interesting to note that TGA-IgA (8.8%) was common in patients with complement deficiencies, especially in patients with hereditary angioedema due to C1-inhibitor deficiency (HAE/C1-inhibitor deficiency) (9.1%) (**Table 7**).

**Table 7:** Distribution of autoantibodies among different categories/types of PIDs.

Category/type of PIDs	Number of patients	RBC antibodies(%)	PA(%)	ASCA(%)	ANCA(%)	TGA(%)	ANA(%)
Immunodeficiencies affecting cellular and humoral immunity	78	11.5	5.1	2.6	3.8	5.1	11.5
SCID	14	14.3	0	0	0	0	0
OS	6	33.3	16.7	0	0	0	0
Leaky SCID	9	22.2	0	0	11.1	11.1	11.1
*bona fide* CID	49	6.1	6.1	4.1	4.1	6.1	16.3
Combined immunodeficiencies with associated or syndromic features	28	14.3	7.1	0	0	17.8	7.1
Wiskott–Aldrich syndrome	6	33.3	0	0	0	16.7	0
DiGeorge syndrome	4	25.0	25.0	0	0	0	0
Ataxia telangiectasia	6	0	0	0	0	0	16.7
Hyper-IgE syndrome	7	0	0	0	0	28.6	0
Other CID with syndromic features	5	20	20	0	0	40	20
Predominantly antibody deficiencies	83	2.4	4.8	2.4	1.2	3.6	6.0
Agammaglobulinemia	14	0	0	0	0	0	0
CVID	42	4.8	4.8	4.8	0	7.1	2.4
Other antibody deficiencies	27	0	7.4	0	3.7	0	14.8
Diseases of immune dysregulation	20	20.0	10.0	25.0	30.0	20.0	30.0
ALPS (Fas deficiency)	3	100	66.7	0	66.7	0	33.3
IPEX syndrome	1	100	0	100	0	100	100
LRBA deficiency	1	0	0	0	0	0	0
Immune dysregulation with colis	6	0	0	16.7	50	0	33.3
Familial hemophagocytic lymphohistiocytosis	3	0	0	0	0	0	33.3
Chediak Higashi	2	0	0	50	0	0	50
Other diseases of immune dysregulation	4	0	0	50	25	75	0
Congenital defects of phagocyte number or function	9	11.1	11.1	22.2	0	11.1	11.1
CGD	4	0	0	50.0	0	25.0	25.0
Congenital neutropenia	3	33.3	33.3	0	0	0	0
Other phagocytic defects	2	0	0	0	0	0	0
Defects of intrinsic and innate immunity	6	0	0	16.7	33.3	0	16.7
STAT1 GOF	2	0	0	0	50	0	50
MSMD	4	0	0	25	25	0	0
Complement deficiencies	68	0	0	2.9	1.5	8.8	7.3
HAE/C1-inhibitor deficiency	55	0	0	3.6	1.8	9.1	7.2
Other complement deficiencies	13	0	0	0	0	7.7	7.7

ALPS, autoimmune lymphoproliferative syndrome; ANA, anti-nuclear antibodies; ANCA, anti-neutrophil cytoplasmic antibodies; ASCA, anti-saccharomyces cerevisiae antibody; CD, celiac disease; CGD, chronic granulomatous disease; CID, combined immunodeficiency; CVID, common variable immunodeficiency; GOF, gain-of-function; HAE, hereditary angioedema; IBD, inflammatory bowel disease; IPEX, immune dysfunction, polyendocrinopathy, enteropathy, LRBA, lipopolysaccharide-responsive and beige-like anchor protein; MSMD, mendelian susceptibility to mycobacterial disease; OS, Omenn Syndrome; PA, platelet-bound antibodies; PID, primary immunodeficiency disease; RBC, red blood cell; SCID, severe combined immunodeficiency; STAT, signal transducer and activator of transcription; TGA, anti-transglutaminase antibody.

Sixty-seven out of the 82 patients (81.7%) with autoimmune manifestations were positive for one or more autoantibodies. All patients with AIHA had positive Coombs test. Twelve out of the 14 patients (85.7%) with immune thrombocytopenia (ITP) had positive PA. Among these patients, six (50%) were positive for PA-IgM, three (25%) for PA-IgM and PA-IgG, two (16.7%) for PA-IgM and PA-IgA and one (8.3%) for PA-IgG. Interestingly, PA-IgM and PA-IgG were present in a patient with autoimmune lymphoproliferative syndrome (ALPS) (due to homozygous mutation in *FAS*) and normal platelet count (188000 per uL). IgM (*n* = 2) and IgG (*n* = 1) anti-neutrophil have been detected by indirect GIFT in three patients with autoimmune neutropenia. The prevalence of ASCA and ANCA among patients with IBD (*n* = 19) were 47.4% and 21.0% respectively. All patients with celiac disease (CD) (*n* = 8) had TGA-IgA, while none of them had TGA-IgG. Among patients with rheumatologic diseases (*n* = 11), six had ANA (54.5%), two were positive for RF (18.2%) while none had ACPA.

**Figure 1:**
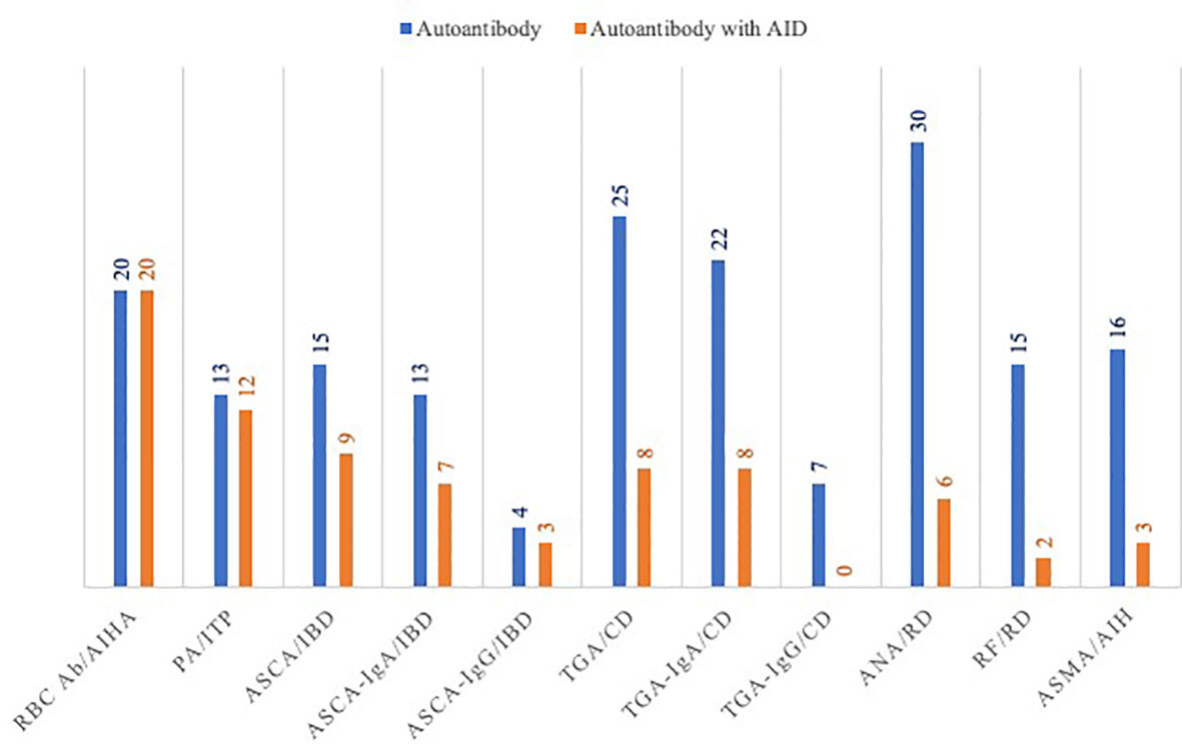
Autoimmune diseases in patients with positive autoantibodies. AIHA, autoimmune hemolytic anemia; ANA, anti-nuclear antibodies; ASCA, anti-saccharomyces cerevisiae antibody; ASMA, anti-smooth muscle antibody; CD, celiac disease; IBD: inflammatory bowel disease; PA, platelet-bound antibodies; RBC Ab, red blood cell antibodies; RD, rheumatoid disease; TGA, anti-transglutaminase antibody.

Almost one third of patients (31%) with positivity for one or more autoantibodies had no autoimmune manifestations. ANA, RF, ASMA, aPL and ANCA were often detected while specific AID was absent (**Figure 1**). Thus, only 20% of patients with positivity for ANA had rheumatologic disease. Such AID was found in only 13.3% of patients with RF. Similarly, 18.7% of patients with positive ASMA had autoimmune hepatitis (AIH) and none of the eight patients who were positive for aPL had anti-phospholipid syndrome (APS).

Eight out of the 22 patients (36.4%) with positive TGA-IgA had CD. The screening of TGA-IgA in our series identified four earlier undiagnosed CD. The diagnostic was confirmed by a biopsy in two patients with CID and CVID and by a “no-biopsy approach” (i.e., TGA-IgA values ≥10 times the upper limit of normal and positive endomysial antibodies (EMA-IgA) in a second serum sample) in two patients with HAE/C1-inhibitor deficiency. Nine out of the 15 patients (60%) with positive ASCA (IgA and or IgG) had IBD. The screening for ASCA identified two undiagnosed IBD in two patients with CGD and CVID.

## Discussion

In the present study, a large series of Algerian patients with PIDs were systematically tested for a broad panel of autoantibodies. The results of autoantibodies screening were correlated to the presence of autoimmune manifestations. Although the occurrence of autoimmunity in patients with PIDs has been previously reported, to the best of our knowledge, this is the first report to evaluate the diagnostic and predictive values of autoantibodies for AIDs in the context of PIDs. Moreover this is the first systematic report of autoimmunity and autoantibody profile in patients with PIDs from Africa.

A total of 299 Algerian patients with 60 different PIDs, belonging to 7 different categories of IUIS classification, were included in this study. Autoimmune manifestations were present in 27.4% of patients. A similar prevalence (26.2%) has been reported in a previous study of the French National Primary Immunodeficiencies Registry (6). However, the frequency of AIDs in the Slovenian (22%) and Kuwaiti (20%) studies were lower (8, 10). Many factors, such as ethnicity, environment, cohort size, age of patients, disease duration, and access to hematopoietic stem cell transplantation could have contributed to such differences between series. In our study, AIDs were more common in patients with immune dysregulation (70.0%) followed by immunodeficiencies affecting cellular and humoral immunity (33.3%). Although autoimmunity was less prevalent in patients with complement deficiencies, it is worth to be mentioned that six out of the 55 patients (10.9%) with HAE/C1-inhibitor deficiency (which is the most common complement deficiency in our series) had autoimmune diseases, including two patients (3.6%) with systemic lupus erythematosus and two others (3.6%) with CD.

Autoimmune cytopenia (10.4%) was the most common autoimmune manifestation in our series. This finding is in accordance with the French and the Kuwaiti studies and emphasize the importance of recognizing autoimmune cytopenia as ‘warning sign’ of immunodeficiency (especially in children) that should trigger an immune evaluation. Furthermore, gastrointestinal disorders (10.0%), rheumatologic diseases (3.7%) and endocrine disorders (3.3%) were also common in our series. This is comparable to the percentages found in the French study (9.5%, 5.0% and 3.2%, respectively) (6, 8). However, skin manifestations, which have been observed in 3.3% of our patients were more frequent in the French registry (5.5%) (6).

In our series, autoantibodies were present in 97 patients (32.4%), they were significantly more frequent than in the control group (*P* < 0.0005). Among patients with positive autoantibodies, 67 (69%) had AIDs while 30 (31%) did not exhibit any autoimmune manifestation. The presence of autoantibodies was a common feature of all types of PIDs. However, their prevalence was significantly higher in patients with defects of immune dysregulation. In our study, all patients with ALPS, lipopolysaccharide-responsive and beige-like anchor protein (LRBA) deficiency and immune dysfunction, polyendocrinopathy, enteropathy, X-linked (IPEX) syndrome developed autoantibodies, highlighting the role of B-cell homeostasis (Fas deficiency), Treg (IPEX syndrome) and cytotoxic T-lymphocyte-associated protein 4 (CTLA-4) surface expression (LRBA deficiency) in the control of autoreactive B cells and autoantibody production. Furthermore, 38.5% of patients with immunodeficiencies affecting cellular and humoral immunity were positive for at least one autoantibody. Although this category is mainly characterized by defects in T-cell development and function (1), aberrant autoantibody production is likely to be related to defects in B-cell tolerance and function. In recombination-activating gene (RAG) deficiency, the presence of a wide spectrum of autoantibodies, both in patients (11) and in animal models (12, 13) has been attributed to defects in central and peripheral B-cell tolerance. Re-expression of RAG proteins in mice allows receptor editing in bone marrow immature B cells and reduce the frequency of self-reactive B-cells (14).

Red blood cell and platelet-bound antibodies were detected in 6.7% and 4.3% of patients respectively. All patients with AIHA had positive Coombs test, while 85.7% of patients with ITP had positive PA. The detection of PA by flow cytometry is an excellent diagnostic tool for ITP, but it is worth noting that PA could be detected in the absence of ITP. Unlike RBC antibodies, most of detected platelet-bound antibodies were IgM. PA-IgM was present in 78.6% of patients with ITP (mostly children), while PA-IgG was detected in only 28.6% of patients. This finding is in accordance with a recent Danish study, in which PA-IgM were significantly more frequent than PA-IgG in children with ITP (63% *vs.* 44%, *P* = 0.03) (15). Schmidt et al have also reported that anti-platelet antibodies were predominantly of the IgM class in children with newly diagnosed ITP (16). In the same study, the authors have noted that IgM responses were present short term without evidence of class-switching to IgG (16). Such responses are likely to be triggered by infections, and molecular mimicry may play a central role in the development of self-directed anti-platelet immunity (17, 18). Furthermore, a pathophysiological role of IgM anti-platelet antibodies in ITP has been demonstrated (16, 19). Unlike IgM anti-erythrocyte autoantibody that promotes anemia through a massive agglutination of RBCs in spleen and liver, PA-IgM induces thrombocytopenia through uptake of opsonized platelets (19, 20). In the mouse, it has been reported that IgM autoantibody-mediated thrombocytopenia was macrophage dependent and the Fcα/μR was required for macrophage uptake of opsonized thrombocytes (21). Complement fixation on IgM might also potentially activate phagocytosis involving complement receptors expressed on macrophages (16). Taken all findings together, childhood ITP unlike chronic adult ITP is mainly IgM-mediated. Patients should be tested for both PA-IgM and PA-IgG in order to improve the sensitivity of the flow cytometric test and to determine the prognosis of the ITP (16).

When considering PID patients with increased susceptibility to infections (i.e., after excluding patients with diseases of immune regulation), RBC antibodies and PA were more common in patients with T-cell immunodeficiency. Of 8 RAG-deficient patients, 3 (37.5%) were positive for RBC antibodies or PA and exhibited autoimmune cytopenia. In previous studies, biallelic *RAG1* and *RAG2* mutations have been associated with a wide range of clinical and immunological phenotypes (22). While null mutations result in SCID with the absence of T and B lymphocytes (T− B− NK+ SCID) (23), hypomorphic mutations allowing for residual protein function are associated with atypical forms including OS (24–26), and leaky SCID (23, 27). Autoimmune manifestations are rare in RAG-deficient patients presenting with typical SCID. However, cytopenias, in particular AIHA, have been reported in more than half of patients with hypomorphic mutations in *RAG1* and *RAG2* (28). In our series, none of the two RAG-deficient patients with SCID had autoimmune cytopenia while three out of the 6 patients with hypomorphic mutations (manifesting as OS in 4 patients and leaky SCID in 2 others) presented with autoimmune cytopenias, including AIHA in two patients and ITP in another one.

Inflammatory bowel disease was the most common gastrointestinal disease in our cohort. IBD was more frequent in patients with defects of immune dysregulation (40%), CGD (50%), and CVID (9.5%). The frequencies of ASCA and ANCA among patients with IBD were 47.4% and 21.0%, respectively. ASCA was associated with IBD in 60% of patients. Interestingly, the systematic testing for ASCA identified two undiagnosed IBD, representing 10.5% of total IBD cases. Hence, patients presenting PIDs with a high risk for IBD, such as CGD and CVID, should be tested for ASCA even in the absence of digestive symptoms. In case of positive ASCA, additional investigations (ex., radiology, colonoscopy and biopsy) are necessary to confirm the diagnosis.

One of the most striking results of our study is the high frequency of TGA. We were somewhat surprised to observe that 25 patients (8.4%) were positive for TGA-IgA and/or TGA-IgG (compared to 1.7% in the control group). Among patients with TGA-IgA, only 8 (36.4%) had CD. Despite their low positive predictive value (PPV), TGA-IgA testing identified four earlier undiagnosed CD, representing 50% of total CD cases in our series. Interestingly enough, the screening of HAE patients for TGA-IgA revealed a frequent association of CD with C1-inhibitor deficiency (3.6%). Such association was also reported by a Hungarian study in which 3.1% of patients with HAE had CD (29). Considering the high prevalence of CD among patients with HAE, screening for the former is warranted. In addition, it may help in differential diagnosis as well as in the selection of the most appropriate therapy, which is very important known the similarities between the symptoms of HAE and CD.

In our study, ANA and RF were present in 10% and 5% of patients respectively. However, their PPVs for rheumatologic diseases were very low (20% and 13.3% for ANA and FR, respectively). Similarly, only 18.7% of patients with positive ASMA had AIH and none of the positive patients for aPL had APS. The aberrant autoantibody production in patients with PIDs may be related to several intrinsic and environmental factors (7, 30). Infections would play a central role in triggering autoimmunity and autoantibody production in patients with PIDs. In fact, the presence of autoantibodies such as ANA, RF, ACPA, aPL, ANCA and ASMA has been described as an epiphenomenon in several bacterial and viral infections (ex., *Staphylococcus aureus*, *Mycobacterium tuberculosis* and *HCV*) (30–34).

In conclusion, the present study provides valuable information about the frequency and the diagnostic/predictive value of a large panel of autoantibodies in PIDs. Considering the frequent association of some AIDs with certain PIDs, such as autoimmune cytopenia/Fas and RAG deficiencies, CD/C1-inhibitor deficiency and IBD/CGD, systematic screening for corresponding autoantibodies would be recommended. However, positivity for autoantibodies, especially ANA, RF, ANCA, aPL and ASMA, should be interpreted with caution due to their low positive predictive value.

## Data Availability Statement

The raw data supporting the conclusions of this article will be made available by the authors, without undue reservation.

## Ethics Statement

The studies involving human participants were reviewed and approved by Local Ethics Committee of Rouiba Hospital, Algiers, Algeria. Written informed consent to participate in this study was provided by the participants’ legal guardian/next of kin.

## Author Contributions

AT: designed the study, conducted flow cytometry assays and autoantibody testing, collected and analyzed data, performed statistical analysis, and wrote the manuscript. AY: contributed to data analysis and interpretation, and edited the manuscript. HI, ST, AS and AO: contributed to autoantibody testing. SL, RBe, SA, LA, DB, SM, CBo, WD, CBe, AK, HMe, NB, AI, TBM, HB, KB, MK, NC, LS, ZA, ZZ, ZB, OI, HMa, and RBo: provided clinical diagnosis of patients. KD: designed and supervised the study, and edited the manuscript. All authors contributed to the article and approved the submitted version.

## Conflict of Interest

The authors declare that the research was conducted in the absence of any commercial or financial relationships that could be construed as a potential conflict of interest.
